# Beyond Phenolics: Alternative Substrates for Type III Copper Enzymes

**DOI:** 10.1002/cbic.202400982

**Published:** 2025-02-18

**Authors:** Matthias Pretzler, Annette Rompel

**Affiliations:** ^1^ Universität Wien Fakultät für Chemie Institut für Biophysikalische Chemie Josef-Holaubek-Platz 2 1090 Wien Austria

**Keywords:** Type III copper enzymes, Tyrosinases, Aromatic amines, Methoxyphenols, Alternative substrate

## Abstract

The type III copper enzyme family of tyrosinases (TYRs) catalyzes the *ortho*‐hydroxylation and oxidation of phenols as well as the two‐electron oxidation of catechols to *ortho*‐quinones. TYRs use copper ions as their tightly bound cofactors and utilize molecular oxygen as their cosubstrate. They are responsible for physiologically important reactions like the formation of melanin, the primary pigment animals apply for protection against UV light. While the reactivity of TYRs on substrates containing aromatic hydroxy groups (*i. e*. phenols) is well recognized, reports clearly demonstrating that TYRs are active on aromatic amines as well have gone largely unnoticed. In this perspective we aim to bring together the sparse data on non‐phenolic TYR substrates to illustrate the potential of TYRs for the oxidation of aminophenols and anilines. The activity of TYRs on aromatic amines extends the substance classes amenable to biotechnological production with TYRs from catechols to *N*‐phenyl imines and phenoxazinone derivates and calls for the inclusion of TYRs among the candidates for oxidative modification of aromatic amines in metabolic pathways.

## Graphical abstract

The copper‐containing metalloenzyme tyrosinase is well known for synthesis of melanin pigments and enzymatic browning of fruits and vegetables. These reactions proceed from phenolics, but tyrosinases can also convert aromatic amines. This perspective brings together the scarce data on the enzymatic conversion of non‐phenolic substrates by these important enzymes.

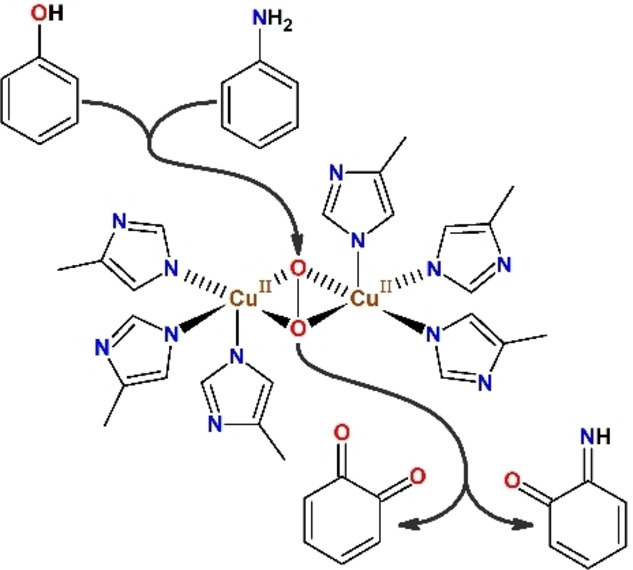

## Biochemistry of Tyrosinases

**Figure 1:**
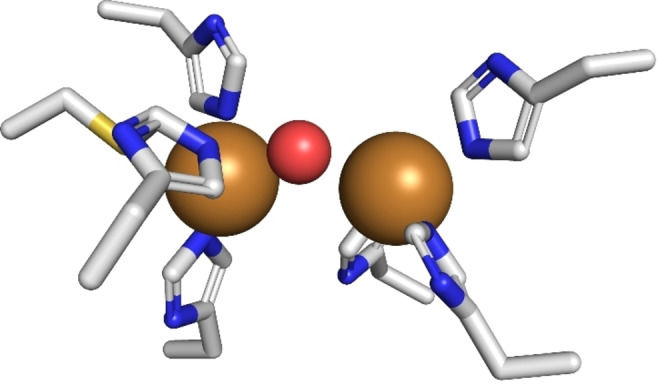
Active site of the fungal TYR *Ab*PPO4 (PDB entry 5M6B).^[2]^ The side chains of the six perfectly conserved copper coordinating histidines are drawn as sticks colored according to the constituent elements (grey: carbon, blue: nitrogen, yellow: sulfur). One of the six histidines shows a thioether bond between the γ‐S of cysteine and C2 of the imidazole ring, a post‐translational modification typical for eukaryotic TYRs.^[3]^ The copper ions themselves are drawn as brown spheres, the oxygen containing molecule (water or a hydroxide ion) coordinated between the two copper ions is shown as a small red sphere. The figure was drawn with PyMOL 2.6 (Schrödinger LLC).

Tyrosinases (TYRs) are a family of copper‐containing oxidases that are present in all domains of life.^[1]^ Their active site is formed by a type III copper center that contains two copper ions, each coordinated by three imidazole‐nitrogens that are provided by six perfectly conserved histidines (Figure 1).

**Scheme 1:**
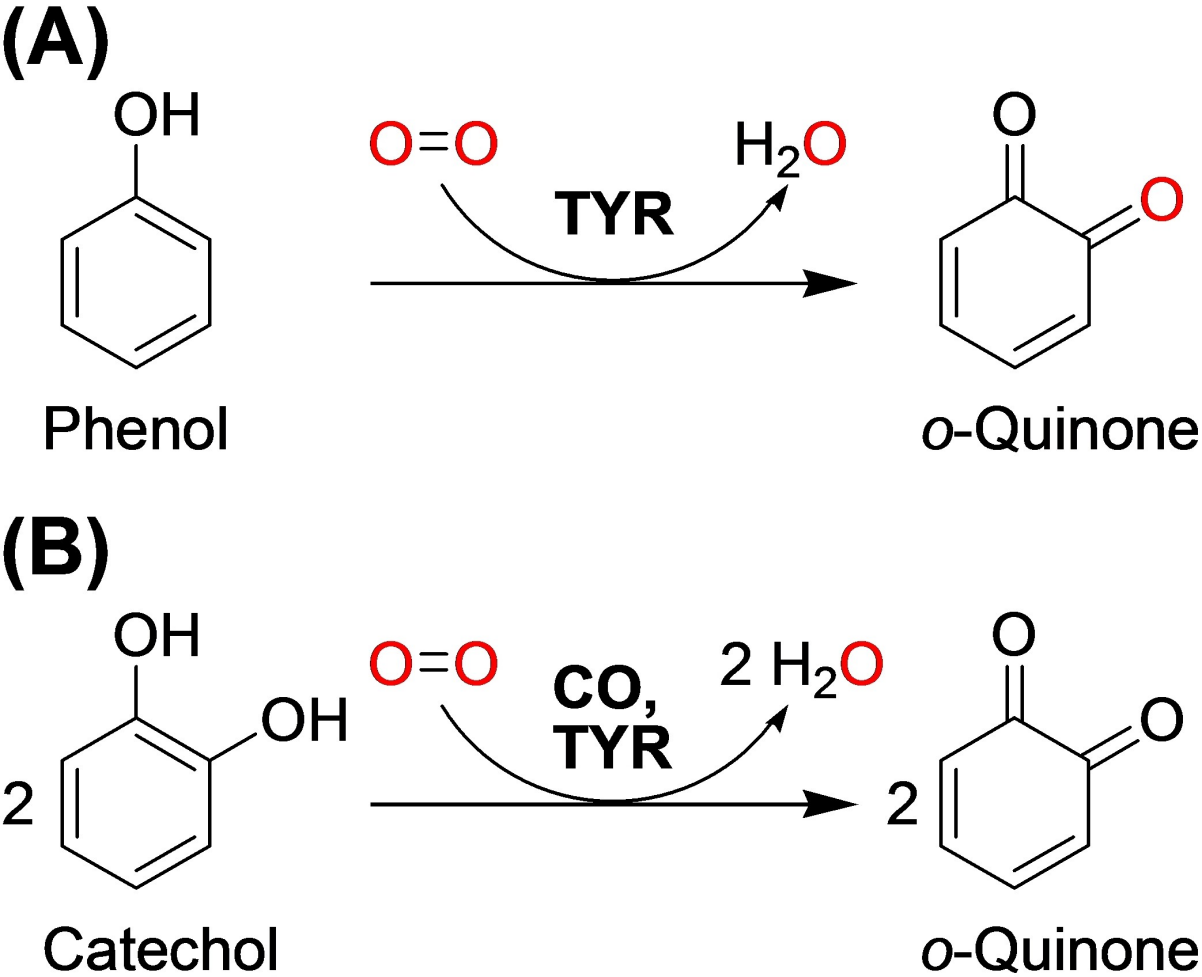
Canonical reactions of (**A**) tyrosinase (TYR, EC 1.14.18.1) and (**B**) catechol oxidase (CO, EC 1.10.3.1) on phenolic substrates.

The canonical reaction of tyrosinases (EC 1.14.18.1) is the *ortho*‐hydroxylation and subsequent oxidation of phenols (tyrosinase or cresolase activity, EC 1.14.18.1) or the two‐electron oxidation of catechols (catechol oxidase or diphenolase activity, EC 1.10.3.1), each yielding the respective *o*‐quinone (Scheme 1).^[4]^ While there are reports of the release of minute amounts of catechol from the active site of TYRs operating on difficult substrates,^[5]^ this effect is likely to be inconsequential for physiological TYR substrates. The TYR reaction requires the presence of copper ions in the enzyme which allows the cosubstrate molecular oxygen to bind to the type III copper center in a characteristic “side‐on” (μ‐η^2^ : η^2^) configuration as a peroxide ion (O_2_^2−^).^[6]^ Hydroxylation of phenolic substrates requires deprotonation of the incoming phenol before it can be coordinated to the active site copper ions. This functionality has been attributed to three of the six copper‐coordinating histidines, which are assisted in abstracting a proton from the incoming substrate by the amino acid side chains next to them.^[7]^ A newly proposed alternative mechanism assigns the role of the initial proton acceptor to the bound peroxide ion^[8]^ (Figure S1).

**Figure 2:**
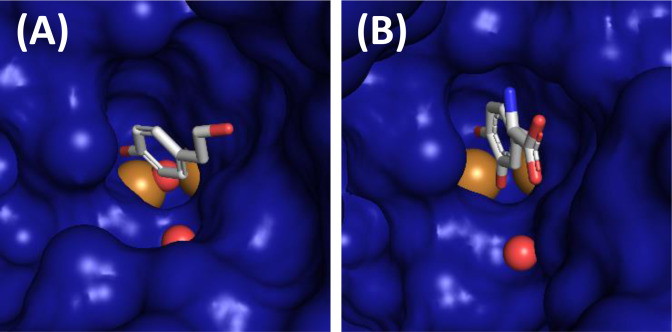
Substrates in the active site pockets of a bacterial and a fungal TYR. (**A**) shows tyrosol bound to the active site of the bacterial TYR *Bm*TYR from *Bacillus megaterium* (PDB‐ID 4P6T).^[9]^ (**B**) depicts already hydroxylated l‐tyrosine in the type III copper center of the fungal TYR *Ao*MelB from *Aspergillus oryzae* (PDB‐ID 6JU9).^[10]^ The solvent accessible surfaces of the proteins are shown metallic blue, the copper ions of the active site are drawn as brown spheres, the small red spheres denote water molecules with positions conserved between crystal structures of different TYRs and the bound substrate molecules are presented as sticks colored according to the constituent elements (grey: carbon, red: oxygen, blue: nitrogen). The figures were prepared with PyMOL 2.6 (Schrödinger LLC).

Productive binding of (phenolic) substrates to the active site of TYRs is believed to involve close proximity of both the hydroxy group of the incoming substrate and the oxygen already bound at the active center (peroxide ion for TYR activity, peroxide or hydroxide/water for CO activity) to the two copper ions of the active center. Currently, six crystal structures containing a freely diffusible small substrate bound to the active center (*Bm*TYR from the bacterium *Bacillus megaterium*: PDB‐IDs 4P6R,^[9]^ 4P6S,^[9]^ 4P6T^[9]^; fungal TYR *Ao*MelB from *Aspergillus oryzae*: 6JU7,^[10]^ 6JU9^[10]^ and the bacterial TYR *Pa*PvdP from *Pseudomonas aeruginosa*: 6EYV)^[11]^ are available. To prepare two thirds of these crystals the enzymatic activity of the crystallized TYRs had to be attenuated by replacing the active site coppers with redox‐inactive zinc ions (4P6R, 4P6S and 6EYV) or removing the metal cofactor altogether (6JU7). The crystal structure of the bacterial *Bm*TYR (PDB‐ID 4P6T, Figure 2A) contains both copper ions and the phenolic substrate *p*‐tyrosol bound to the active site. Interestingly, the electron density of this structure does indeed indicate hydroxylation of the bound substrate at the carbon next to the hydroxy group.^[9]^ The same observation was made with the crystal structure prepared from the Cys92 to Ala mutant (removal of the thioether bond) of the fungal TYR *Ao*MelB containing the prototypical TYR substrate l‐tyrosine (PDB‐ID 6JU9, Figure 2B). Also in this structure hydroxylation at the *ortho*‐position of the aromatic ring next to the hydroxyl group was discernable in the electron density.^[10]^

The speed of the catalyzed reaction does depend on the substrate, the used enzyme and the reaction conditions. Reaction rates of TYRs do vary considerably with reported reaction speeds (*k_cat_*‐values, *i. e*. conversion events per protein chain) of one converted molecule per 85 s to 320 molecules per second for monophenols and one oxidation every 25 s to 7220 oxidation events per second for catechols (data from the BRENDA enzyme database).^[12]^

**Scheme 2:**
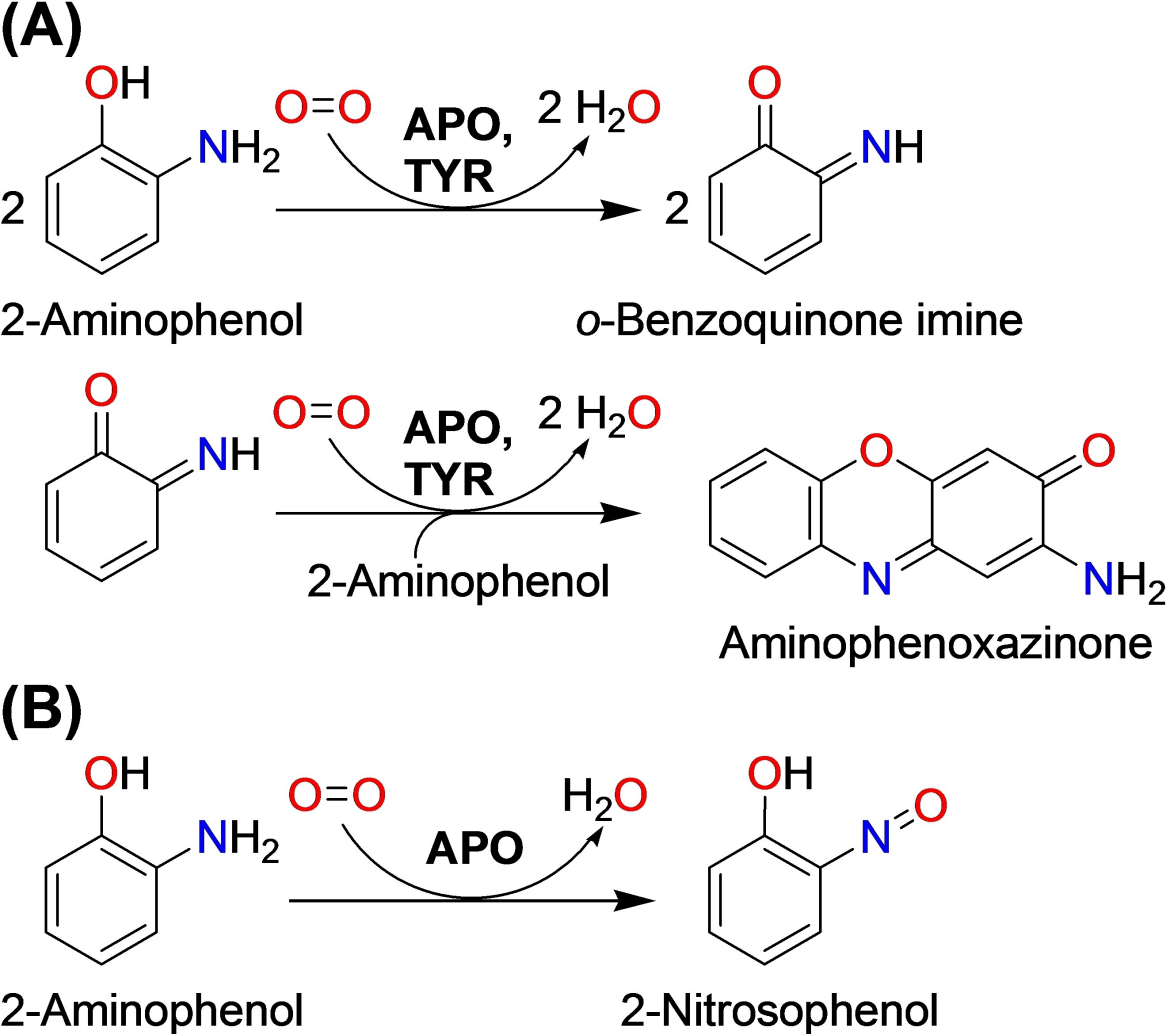
Reactions catalyzed by *o*‐aminophenol oxidases (APO, EC 1.10.3.4). (**A**) shows the reactivity of APOs on *ortho*‐aminophenols. The formed *o*‐quinone imines do usually form adducts with unreacted *o*‐aminophenols, which leads to the formation of (often brightly colored) phenoxazinones. This reaction is also catalyzed by many TYRs,^[17]^ while the formation of the nitroso product shown in (**B**) is limited to APOs.^[14]^ The identities of the 2‐nitrosophenols formed by enzymatic action of *Sm*NspF on 3‐amino‐4‐hydroxybenzoic acid or 3‐amino‐4‐hydroxybenzaldehyde were established by ^1^H‐ and ^13^C‐NMR as well as mass spectrometry.^[14]^

Oxidation of mixed oxygen‐nitrogen substrates by either two‐electron oxidation (Scheme 2A) or monooxygenation of the amino group (Scheme 2B) has been described for the TYR subtype of *o*‐aminophenol oxidases (APOs, EC 1.10.3.4). Data on this class of enzymes is rare as to date only two bacterial APOs have been characterized: *Sg*GriF, which is encoded within the grixazone biosynthesis gene cluster of the bacterium *Streptomyces griseus*,^[13]^ and the closely related *Sm*NspF that catalyzes the final oxidation necessary for the biosynthesis of 4‐hydroxy‐3‐nitrosobenzamide in *Streptomyces murayamaensis*.^[14]^ The two‐electron oxidation that converts aminophenols into phenoxazinones is also catalyzed by phenoxazinone synthases (also EC 1.10.3.4),^[15]^ which are not TYRs but laccases. They contain a type III copper center which together with a spatially very close type II copper center forms the typical trinuclear copper cluster of laccases where oxygen is reduced to water. In addition, laccases contain a type I copper center that is responsible for oxidation of their substrates by single electron abstraction.^[16]^

## Hydroxylation and Oxidation of Anilines

Acceptance of non‐phenolic substances as TYR substrates was first shown in 1961 for *Nc*TYR from the sac fungus *Neurospora crassa*.^[18]^ This enzyme catalyzed the oxidation of 0.36 mM 1,4‐diaminobenzene (Structures of all substrates mentioned in this text are presented in Figure S2.) at 2.8 % of the rate on 0.036 mM of the catechol l‐DOPA.^[18]^

**Scheme 3:**
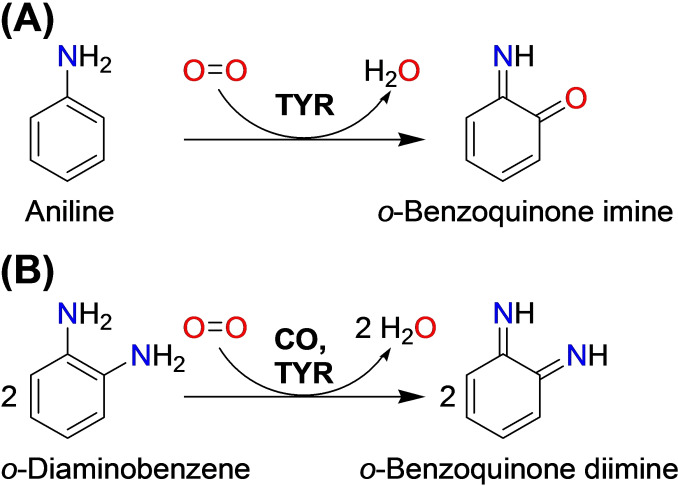
Hydroxylation and oxidation of aromatic amines by TYRs.

**Scheme 4:**
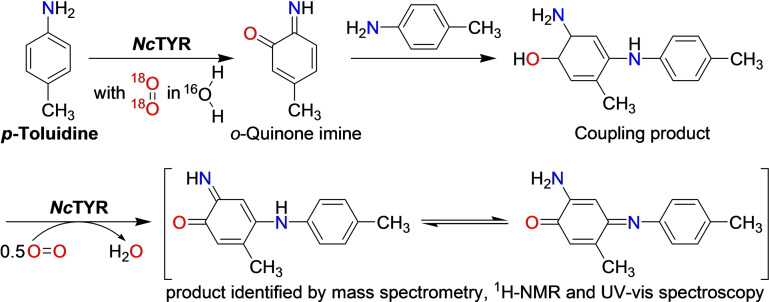
Hydroxylation and oxidation of *p*‐toluidine by *Nc*TYR.^[17]^

The first direct comparison of kinetic constants with anilines and the corresponding phenols was also prepared with *Nc*TYR, showing an approximately 1000‐fold slower reaction on the anilines compared to the respective phenols^[17]^ (Table 1). The product of the reaction of *Nc*TYR on *p*‐toluidine (Scheme 4) was identified by means of ^18^O‐labeling, mass spectrometry, ^1^H NMR and UV‐vis spectroscopy as being derived from an *o*‐quinone imine.^[17]^ Chemical oxidation of *p*‐toluidine with Frémy's salt (potassium nitrosodisulfonate) yielded the same product.^[29]^ Reactivity on non‐phenolic substrates has been reported for TYRs from four fungi and for the plant enzyme *Si*PPO from black sesame (Table 2). Aromatic amines make up the majority of non‐phenolics reported as TYR substrates (Scheme 3), there is a single report each for acceptance of an indole^[19]^ and of thiols^[20]^ as TYR substrates (*vide infra* for details and Figure S3 for their structures). In addition, activity on *ortho*‐dimethoxylated phenols was shown for four short (*i. e*. single domain TYRs, Figure S4) fungal TYRs.^[21]^ Activity on *ortho*‐monomethoxylated phenolic substrates is encompassed by the canonical TYR mechanism and therefore is not considered here.

**Table 1:** Kinetic constants of *Nc*TYR on *o*‐aminophenols, anilines and the corresponding catechols or phenols.^[17]^

*o*‐Aminophenol	*k_cat_*/s^−^1	*K_M_*/mM	Catechol	*k_cat_*/s^−1^	*K_M_*/mM
2‐amino‐3‐hydroxybenzoic acid	0.25±0.01	0.7±0.1	2,3‐dihydroxybenzoic acid	0.18±0.01	3.3±0.1
3‐amino‐2‐hydroxybenzoic acid	0.40±0.01	11.3±0.1			
3‐amino‐4‐hydroxybenzoic acid	0.83±0.01	4.5±0.1	3,4‐dihydroxybenzoic acid	51.4±0.2	2.7±0.1
4‐amino‐3‐hydroxybenzoic acid	0.60±0.01	4.4±0.1			

Aromatic *o–*diamine	*k_cat_/*s^−1^	*K_M_*/mM	Catechol	*k_cat_/*s^−1^	*K_M_*/mM
3,4‐diaminobenzoic acid	0.60±0.01	2.1±0.1	3,4‐dihydroxybenzoic acid	51.4±0.2	2.7±0.1
Aniline	*k_cat_/*s^−1^	*K_M_*/mM	Phenol	*k_cat_/*s^−1^	*K_M_*/mM
4‐aminophenylalanine	0.050±0.002	0.37±0.02	4‐hydroxyphenylalanine (tyrosine)	116±1	0.34±0.02
4‐aminotoluene (*p*‐toluidine)	0.025±0.002	0.11±0.02	4‐hydroxytoluene (*p*‐cresol)	42±0.5	0.13±0.02
4‐aminoacetanilide	0.033±0.002	0.18±0.02	4‐hydroxyacetanilide	22±0.9	0.49±0.02

Reaction conditions: 0.1 M potassium phosphate buffer pH 7 (3 ml), substrate concentration range 0.24–24 mM; temperature 24 °C. For *k_cat_/K_M_* values please refer to Table S1.

**Table 2:** TYRs with reported activity on non‐standard substrates.

Enzyme	Host organism	UniProt ID	Ref.
*Ab*PPO1	*Agaricus bisporus*	OU703638^[a]^	[4]
*Ab*PPO2	*Agaricus bisporus*	O42713	[4]
*Ab*PPO3	*Agaricus bisporus*	C7FF04	[4]
*Ab*PPO4	*Agaricus bisporus*	C7FF05	[4]
*Ab*PPO5	*Agaricus bisporus*	OU703645^[a]^	[4]
*Ab*PPO6	*Agaricus bisporus*	OU703647^[a]^	[4]
*Ab*T	*Agaricus bisporus*	–^[b]^	[22–24]
*Ao*CO4	*Aspergillus oryzae*	Q2UNF9	[25]
*Cg*PPO1^[c]^	*Chaetomium globosum*	Q2H7I7	[21]
*Cg*PPO2^[c]^	*Chaetomium globosum*	Q2GZJ4	[21]
*Mt*PPO7^[c]^	*Thermothelomyces thermophila*	G2QC95	[21]
*Nc*TYR	*Neurospora crassa*	P00440	[17,18]
*Si*PPO	*Sesamum indicum*	A0A6I9UJ96	[19]
*Tr*TYR2	*Trichoderma reesei*	A0ZXZ4	[26]
*Tt*PPO^[c]^	*Thermothelomyces thermophila*	G2QLD3	[21,27]

[a] No UniProt entry is available, the NCBI‐ID for the GenPept database is provided instead. [b] *Ab*T (“mushroom tyrosinase”) is a preparation containing multiple TYR isoforms, the presence of *Ab*PPO3 and *Ab*PPO4 was confirmed.^[28]^ [c] For these short fungal TYRs reactivity on *ortho*‐dimethoxylated phenols was demonstrated but data on their acceptance of anilines is not available yet.

The second enzyme for which the reactivity on anilines and phenols was compared is “mushroom tyrosinase” (*Ab*T), a TYR preparation derived from the common button mushroom *Agaricus bisporus* that is by far the most commonly used source of TYR activity for both basic and applied research. This preparation does not represent a single enzyme but contains mainly two of the six TYR isoenzymes of *A. bisporus*:^[4]^ *Ab*PPO3 and *Ab*PPO4.^[28]^ As was the case for the fungal *Nc*TYR also *Ab*T showed a markedly slower reaction on anilines,^[23]^ but for this enzyme the reduction in *k_cat_* going from phenols to anilines was closer to 100‐fold (Table 3) than to the 1000‐fold reported for *Nc*TYR. Specifically, the *k_cat_* reduction of *Nc*TYR on tyrosine *vs*. 4‐aminophenylalanine is 2320‐fold while the same ratio for *Ab*T is 812. For *p*‐cresol *vs. p*‐toluidine the *k_cat_* ratio of *Nc*TYR is 1680 while for *Ab*T it is only 16.

**Table 3:** Kinetic constants of *Ab*T on aminophenols, anilines and the corresponding catechols or phenols.^[23]^

*o*‐Aminophenol	*k_cat_*/s^−1^	*K_M_*/mM	Catechol	*k_cat_*/s^−1^	*K_M_*/mM
2‐amino‐3‐hydroxybenzoic acid	0.13±0.01	2.27±0.22	2,3‐dihydroxybenzoic acid	0.37±0.04	1.02±0.12
3‐amino‐2‐hydroxybenzoic acid	0.13±0.01	0.36±0.04			
3‐amino‐4‐hydroxybenzoic acid	0.45±0.01	0.11±0.01	3,4‐dihydroxybenzoic acid	8.11±0.31	0.07±0.01
4‐amino‐3‐hydroxybenzoic acid	0.50±0.01	0.15±0.01			
3‐amino‐4‐hydroxytoluene	29.42±1.58	2.73±0.56	4‐methylcatechol	842.12±26.11	0.10±0.01
4‐amino‐3‐hydroxytoluene	26.12±2.13	2.36±0.43			
3‐amino‐L‐tyrosine	1.35±0.15	0.90±0.18	L–DOPA	102.62±19.21	0.55±0.08
2‐aminophenol	60.57±2.84	0.78±0.08	catechol	874.10±30.21	0.16±0.01

Aromatic *o–*diamine	*k_cat_/*s^−1^	*K_M_*/mM	Catechol	*k_cat_/*s^−1^	*K_M_*/mM
2,3‐diaminobenzoic acid	0.05±0.01	0.28±0.04	2,3‐dihydroxybenzoic acid	0.37±0.04	1.02±0.12
3,4‐diaminobenzoic acid	0.08±0.01	0.15±0.02	3,4‐dihydroxybenzoic acid	8.11±0.31	0.07±0.01
2,3‐diaminotoluene	0.11±0.01	0.96±0.11	3‐methylcatechol	104.2±4.7	1.10±0.14
3,4‐diaminotoluene	0.51±0.04	0.06±0.01	4‐methylcatechol	842.12±26.11	0.10±0.01
4‐methoxy‐1,2‐phenylendiamine	1.51±0.13	0.36±0.05	3‐methoxycatechol	17.83±0.53	1.26±0.13
1,2‐diaminobenzene	0.53±0.05	0.46±0.05	catechol	874.10±30.21	0.16±0.01

Aniline	*k_cat_/*s^−1^	*K_M_*/mM	Phenol	*k_cat_/*s^−1^	*K_M_*/mM
aniline	0.45±0.03	0.64±0.09	phenol	10.81±1.51	0.61±0.04
2‐aminobenzoic acid	0.00013±0.00001	2.36±0.34	2‐hydroxybenzoic acid	0.046±0.002	2.62±0.18
3‐aminobenzoic acid	0.00033±0.00001	0.28±0.02	3‐hydroxybenzoic acid	0.64±0.03	2.35±0.32
4‐aminobenzoic acid	0.00504±0.00032	0.14±0.01	4‐hydroxybenzoic acid	1.70±0.07	0.22±0.03
2‐aminotoluene	0.33±0.02	2.93±0.38	2‐methylphenol	7.37±0.40	1.48±0.18
3‐aminotoluene	0.46±0.04	1.26±0.07	3‐methylphenol	7.51±0.65	0.62±0.09
4‐aminotoluene (*p‐*toluidine)	1.01±0.03	1.04±0.12	4‐methylphenol (*p*‐cresol)	16.12±1.53	0.38±0.05
2‐aminoanisole	0.09±0.01	0.26±0.03	2‐hydroxyanisole	5.47±0.21	5.40±0.60
3‐aminoanisole	0.12±0.01	0.15±0.02	3‐hydroxyanisole	46.87±2.06	2.53±0.26
4‐aminoanisole	1.24±0.21	0.09±0.01	4‐hydroxyanisole	184.20±6.10	0.08±0.01
4‐aminophenylalanine	0.01±0.01	0.42±0.05	4‐hydroxyphenylalanine (tyrosine)	8.12±1.44	0.27±0.03

Reaction conditions: 0.06–0.9 μM *Ab*T in 30 mM sodium phosphate buffer (pH 7.0) with suitable concentrations of either the reductant ascorbic acid or reduced nicotinamide adenine dinucleotide (NADH), the nucleophile 3‐methyl‐2‐benzothiazolinonehydrazone (MBTH) or no reagent addition; temperature 25 °C. For *k_cat_/K_M_* values please refer to Table S2.

Using tyrosinase isolated from fresh *A. bisporus* fruiting bodies the ability of *Ab*T for hydroxylation and oxidation of 4‐aminophenylalanine and the oxidation of 3,4‐diaminobenzoic acid and 3,4‐diaminoanisole was confirmed in an independent study.^[22]^ In the same study also 3‐aminobenzoic acid and 4‐aminobenzoic acid were tested as substrates of *Ab*T, but no activity was measured. As shown in Table 3 these two substrates are poor substrates for *Ab*T, so the two aminobenzoic acids most likely did not meet the threshold for confirmation of activity employed in this study.^[22]^

Unsubstituted aniline is a substrate for two other fungal TYRs: Both *Tr*TYR2 from *Trichoderma reesei* ^[26]^ and the extracellular short TYR *Ao*CO4 from *Aspergillus oryzae* ^[25]^ were able to use aniline as substrate.

The latest report of TYRs active on aromatic amines comes again from *A. bisporus*. Four of the six isoenzymes of mushroom tyrosinase (*Ab*PPO1‐4) were shown to be active on *p*‐toluidine while all six *A. bisporus* TYRs (*Ab*PPO1‐6) were efficient catalysts for the oxidation of 1,2‐diaminobenzene.^[4]^

Currently, there is no report on the acceptance of an aromatic amine as substrate for any non‐fungal TYR.

## Hydrazines

*Ab*T (“mushroom tyrosinase”) was shown to catalyze a peculiar reaction on aromatic hydrazines (Table 4). In this study the authors also showed formation of phenol from phenylhydrazine during the catalysis by the TYR.^[24]^ The formed phenol was identified by HPLC, ESI mass spectrometry and its reaction with the Gibbs reagent (2,6‐dichloroquinone‐4‐chloroimide). The Gibbs reagent reacts with phenol to the oxidized redox indicator 2,6‐dichlorophenolindophenol, which indicates the presence of phenolic molecules by its intense blue color. After consumption of most of the phenylhydrazine, which acts as a reductant, the formed phenol was further hydroxylated and oxidized by the fungal TYR to *o*‐benzoquinone.^[24]^ In addition to the (expected) consumption of oxygen during the enzymatic conversion of phenylhydrazine, formation of appreciable amounts of superoxide (O_2_^−^: 0.96±0.03 μM min^−1^ during the formation of 6.20±0.09 μM min^−1^ of phenol) was detected and the reaction on phenylhydrazine could also be speed up by the addition of the superoxide‐converting enzyme superoxide dismutase from horseradish (EC 1.15.1.1: 2 O_2_^−^+2 H^+^→O_2_+H_2_O_2_).^[24]^ Hydrazine can be oxidized by molecular oxygen and this oxidation reaction is accelerated considerably in the presence of free copper ions.^[30]^ The reported reactions on aromatic hydrazines may best be explained in terms of copper, rather than TYR, being responsible for the observed reactions.

**Table 4:** Kinetic constants of *Ab*T on three aromatic hydrazine derivates.^[24]^

Aromatic hydrazine	*k_cat_*/s^−1^	*K_M_*/mM
phenylhydrazine	11.0±0.3	0.30±0.01
1‐acetyl‐2‐phenylhydrazine	5.0±0.2	3.60±0.11
2,4‐dimethylphenylhydrazine	2.5±0.4	0.29±0.01

Reaction conditions: 0.221 μM *Ab*T in 20 mM 4‐(2‐hydroxyethyl)‐1‐piperazineethanesulfonic acid (HEPES) pH 6.8, substrate concentration range 0.1–1.6 mM; temperature 25 °C.

## Methoxyphenols

The extracellular short fungal TYR *Tt*PPO from the sac fungus *Thermothelomyces thermophila* was shown to be able to oxidize 2,6‐dimethoxyphenol,^[27]^ a phenolic substrate blocked at both *ortho*‐positions that are the targets of the *ortho‐*hydroxylation activity of TYRs.^[7]^ Activity on the methoxyphenol 2‐methoxycinnamic acid was reported for the plant TYR *Si*PPO from *Sesamum indicum* (black sesame was used),^[19]^ but no mechanistic investigations were carried out in this study. In sharp contrast to that report, 2‐methoxycinnamic acid has been established as a competitive inhibitor of mushroom tyrosinase (*Ab*T).^[31]^ The same study also claims activity of *Si*PPO on indole‐3‐carbonic acid^[19]^ (Figure S3), which would be a completely new mode of reaction for TYRs. However, no data to substantiate that claim is presented so TYR activity on indole‐3‐carbonic acid remains speculative.

A similar reaction on a blocked phenol was demonstrated for *Ab*T and five of the six isoenzymes of mushroom tyrosinase (*Ab*PPO1–4 and *Ab*PPO6), which were able to use O‐phospho‐l‐tyrosine as substrate,^[4]^ putatively by cleaving off the phosphate group from the phenolic hydroxy group.

**Scheme 5:**
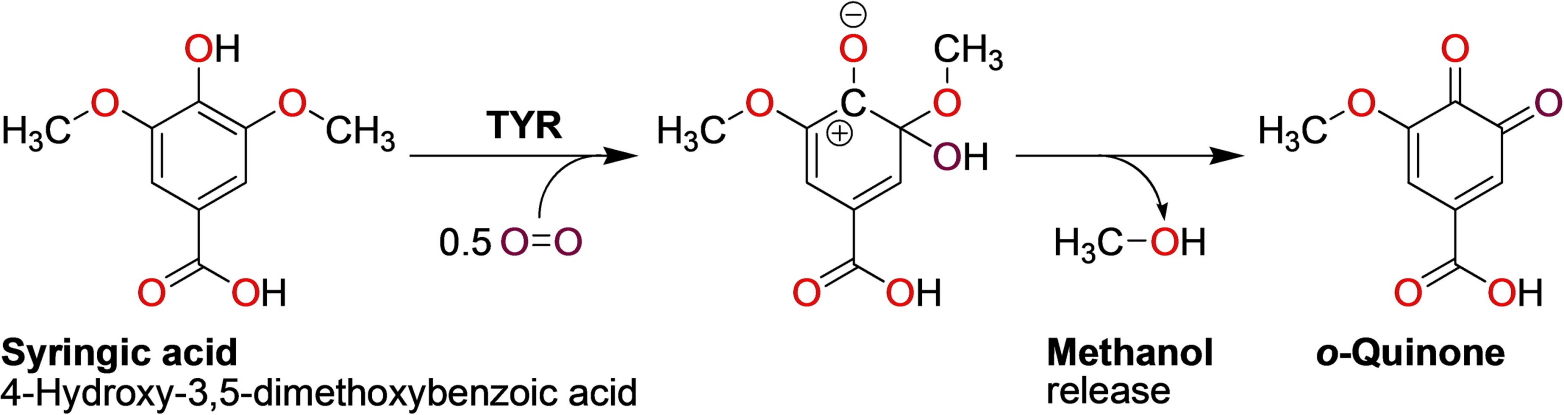
Hydroxylation and oxidation of syringic acid by the short fungal TYR *Mt*PPO7 from *Thermothelomyces thermophila*.^[21]^ The different shades of red used for the oxygens in syringic acid versus the incoming molecular oxygen serve only to illustrate the trajectory of the enzymatically introduced oxygen and do not indicate a chemical difference between these oxygens.

The mechanism of the TYR‐catalyzed oxidation of *ortho*‐methoxyphenols (Scheme 5) was elucidated only recently and proceeds by cleaving off the *ortho*‐methoxy group as methanol after the TYR has added a hydroxy group to the same carbon in *ortho*‐position on the aromatic ring.^[21]^ The incorporation of oxygen from the added molecular oxygen into the oxidized product was demonstrated using isotopically labelled molecular oxygen (^18^O_2_) and mass spectrometric detection of the reaction product after separation of the reaction mixture by high performance liquid chromatography for reactions with vanillic or syringic acid. The presence of methanol in the reaction mixture after TYR had been added to the methoxylated substrate was shown conclusively with a coupled enzyme assay employing alcohol oxidase from *Pichia pastoris* (EC 1.1.3.13, here converting methanol to formaldehyde) and formaldehyde dehydrogenase from *Pseudomonas sp*. (EC 1.2.1.46, used for the oxidation of formaldehyde to formic acid coupled to the reduction of nicotinamide adenine dinucleotide [NAD^+^ to NADH], which was quantified photometrically at 340 nm).

## Thiols

Thiols are potent nucleophiles that readily form adducts with *o*‐quinones.^[32]^ There is a single report that claims the formation of disulfide bridges linking both aromatic and aliphatic thiols by the enzymatic action of “mushroom tyrosinase” (*Ab*T),^[20]^ but the same reaction also reportedly worked with horseradish peroxidase (EC 1.11.1.7) in the absence of hydrogen peroxide. In addition, the used preparation of “mushroom tyrosinase” (*Ab*T) is known to contain a number of contaminating enzymes including laccase,^[33]^ for which thiol‐oxidase activity (EC 1.8.3.2) has been shown.^[34]^ In light of these ambiguities, and the reported ability of free copper ions to catalyze the same reaction *via* a radical mechanism,^[35]^ we believe that the claim of a specifically TYR‐induced coupling of thiols cannot be considered a convincing explanation for the observed formation of disulfides.

## Outlook

Besides phenols, TYRs are also active on aromatic amines, albeit the reaction proceeds significantly slower. These lower reaction rates suggest that substrate deprotonation (pK_a_ of phenols≈10, pK_a_ of anilines ≈25) contributes to the rate determining step of the TYR reaction. The higher acidity of thiols would help in testing this supposition, but their strong reducing properties seem to preclude their use as TYR substrates.

While the name “tyrosinase” may seem at odds with their activity towards aromatic amines, there is currently no type III copper enzyme known that would not preferably accept phenolic substrates. As long as this is the case we believe that the well‐established name “tyrosinase”^[36]^ takes precedence and these reactions are better categorized as alternative activities of TYRs. The reaction rates on anilines of the TYRs presented in this manuscript are quite low, but data for *Ab*PPO1 and *Ab*PPO2 from *Agaricus bisporus* on *p*‐toluidine^[4]^ strongly suggests that this is not a principal limitation.

Further experiments with a larger number of TYRs (including non‐fungal enzymes) and substrates as well as mutation studies aimed at increased reaction rates on aromatic amines will be required in order to harness the so far severely underappreciated reactivity of TYRs on anilines for use in biotechnology (*e. g*. as the active agents in bioremediation, for the organic synthesis of phenoxazinones or other aniline‐based compounds, as industrial catalysts or novel building blocks for synthetic biology) and improved understanding of the metabolism of aromatic amines.

## Supporting Information Summary

The authors have cited additional references within the Supporting Information (Ref. [37–40]).

## Conflict of Interests

The authors declare no conflict of interest.

1

## Supporting information

As a service to our authors and readers, this journal provides supporting information supplied by the authors. Such materials are peer reviewed and may be re‐organized for online delivery, but are not copy‐edited or typeset. Technical support issues arising from supporting information (other than missing files) should be addressed to the authors.

Supporting Information

## Data Availability

Data sharing is not applicable to this article as no new data were created or analyzed in this study.
