# Supplementary material for: Beyond Phenolics: Alternative Substrates for Type III Copper Enzymes
Source: Chembiochem. 2025 Feb 18;26(7):e202400982. doi: 10.1002/cbic.202400982 (PMC12002105; doi:10.1002/cbic.202400982)
Supplement: Supplementary file 1 — Supporting Information [file CBIC-26-e202400982-s001.pdf]

ChemBioChem

Supporting Information

Beyond Phenolics: Alternative Substrates for Type III Copper Enzymes

Matthias Pretzler and Annette Rompel*

Supporting Information
©Wiley-VCH 2025
69451 Weinheim, Germany

Beyond Phenolics: Alternative Substrates for Type III Copper Enzymes

Matthias Pretzler and Annette Rompel*

*correspondence to: annette.rompel@univie.ac.at

Abstract: The type III copper enzyme family of tyrosinases (TYRs) catalyzes the *ortho*-hydroxylation and oxidation of phenols as well as the two-electron oxidation of catechols to *ortho*-quinones. TYRs use copper ions as their tightly bound cofactors and utilize molecular oxygen as their cosubstrate. They are responsible for physiologically important reactions like the formation of melanin, the primary pigment animals apply for protection against UV light. While the reactivity of TYRs on substrates containing aromatic hydroxy groups (*i.e.* phenols) is well recognized, reports clearly demonstrating that TYRs are active on aromatic amines as well have gone largely unnoticed. In this perspective we aim to bring together the sparse data on non-phenolic TYR substrates to illustrate the potential of TYRs for the oxidation of aminophenols and anilines. The activity of TYRs on aromatic amines extends the substance classes amenable to biotechnological production with TYRs from catechols to *N*-phenyl imines and phenoxazinone derivates and calls for the inclusion of TYRs among the candidates for oxidative modification of aromatic amines in metabolic pathways.

DOI: 10.1002/cbic.202400982

Content | page

Fig. S1. Mechanism of TYR acting on monophenols according to Kipouros *et al.* 2

Fig. S2. Structures of the TYR-substrates mentioned in the text. 3 – 6

Fig. S3. Structures of three potential TYR-substrates. 6

Fig. S4. Domain architecture of short fungal tyrosinases. 7

Table S1. Kinetic efficiencies of *Nc*TYR on *o*-aminophenols, anilines and the corresponding catechols or phenols. 8

Table S2. Kinetic efficiencies of *Ab*T on *o*-aminophenols, anilines and the corresponding catechols or phenols. 8

Author contributions 8

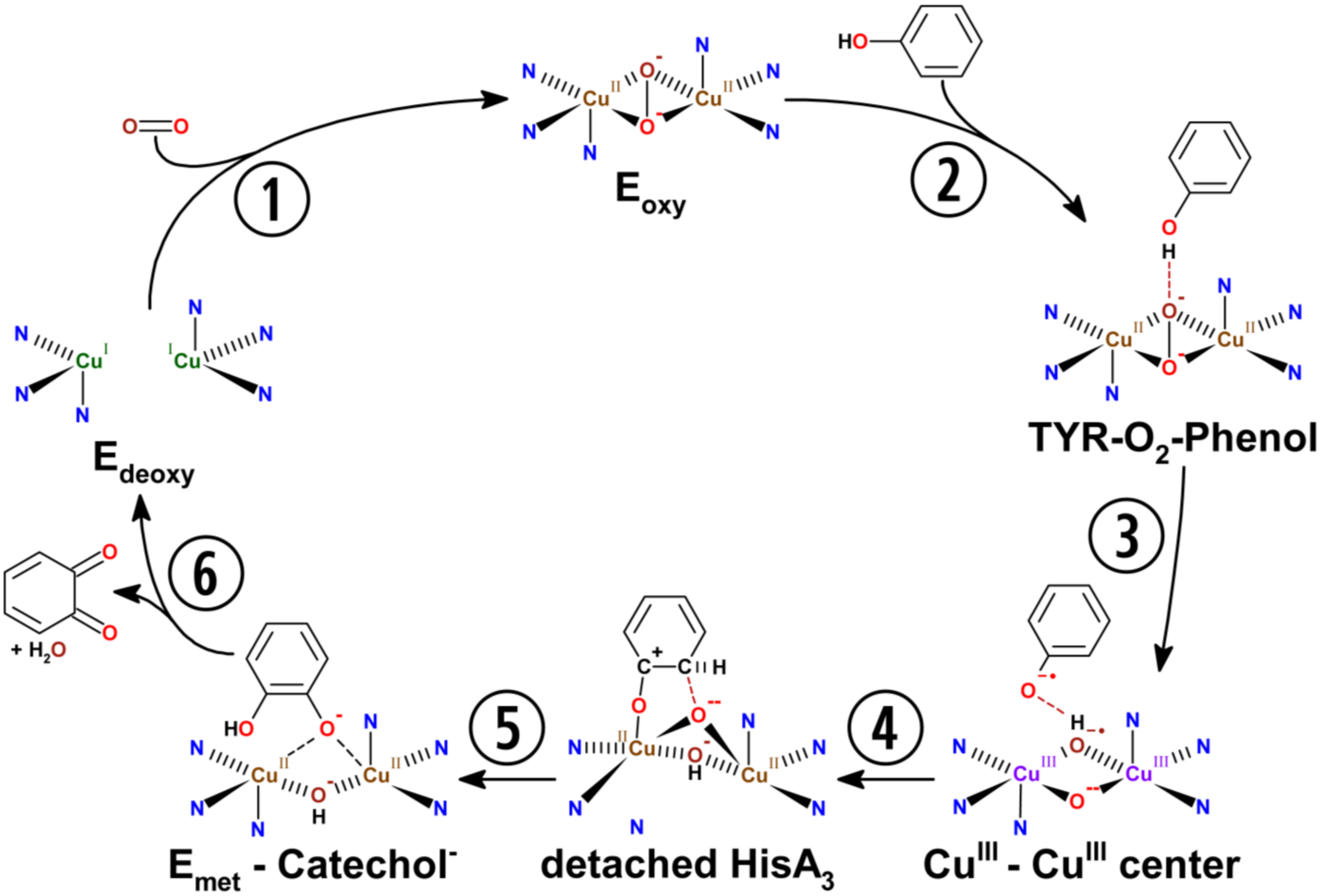

Fig. S1. Mechanism of TYR acting on monophenols according to Kipouros *et al.*[37]
① Binding of molecular oxygen to the reduced type III copper (**E**$_{\mathbf{deoxy}}$) center leads to formation of the oxy form of the enzyme (**E**$_{\mathbf{oxy}}$) in which oxygen is bound as a bridging peroxide (μ-η^2:η^2-O_2^{2-}). ② Coordination of a phenol forms the ternary TYR-O_2-monophenol complex[38]. ③ $H^{+/\bullet}$-transfer from the monophenol to the μ-η^2:η^2-peroxide of E_{oxy} leads to cleavage of the O-O bond and formation of an Cu^{III}/Cu^{III} core[8]. Note that this center is presented as Cu^{III}-$Cu^{II/III}$ in the proposed mechanism[37] but is shown here with the two Cu^{III} states dictated by charge conservation. ④ C-O bond formation is driven by the attack of the non-protonated oxide ion (μ-O^{2-}) on the *ortho*-C of the phenolic substrate. The CuA ion detaches from its axial ligand $HisA_3$ in order to move (≈ 1.6 Å in *S. glaucescens* TYR) towards the phenol and coordinate it[38]. ⑤ The bound catecholate is then oxidized in a two-electron oxidation and the resulting *o*-quinone ⑥ leaves the active site together with water formed from the bridging hydroxide ion (OH^-). The ionization states of the copper ions are marked using green for Cu(I), brown for Cu(II) and violet for Cu(III). The different shades of red used for depiction of the oxygen molecule bound to the active site have no physical meaning but should only help to distinguish between the μ-oxo that takes over the phenolic proton and the other μ-oxo which is responsible for *o*-hydroxylation of the phenol.

Phenols		
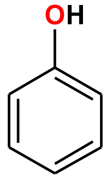<br>**Phenol**<br>Hydroxybenzene<br>Carbolic acid	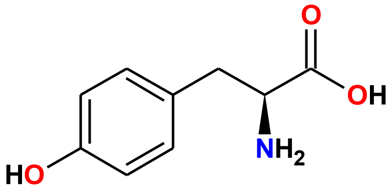<br>**L-Tyrosine**<br>4-Hydroxy-L-phenylalanine	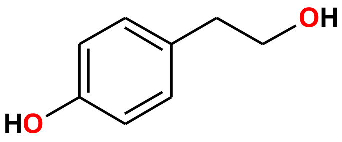<br>**Tyrosol**<br>2-(4-Hydroxyphenyl)ethanol<br>4-(2-Hydroxyethyl)phenol
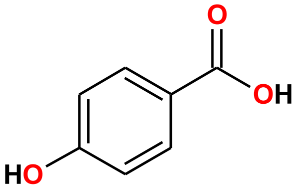<br>**4-Hydroxybenzoic acid**<br>*p*-Salicylic acid<br>4-Carboxyphenol	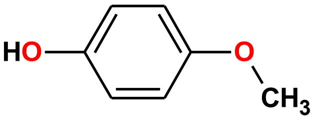<br>**Mequinol**<br>4-Methoxyphenol<br>4-Hydroxyanisole	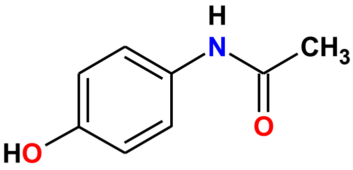<br>**Acetaminophen**<br>Paracetamol<br>4-Hydroxyacetanilide
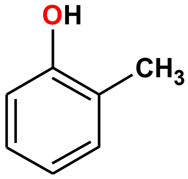<br>***o*-Cresol**<br>2-Methylphenol<br>2-Hydroxytoluene	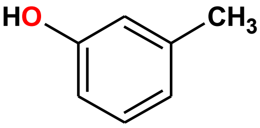<br>***m*-Cresol**<br>3-Methylphenol<br>3-Hydroxytoluene	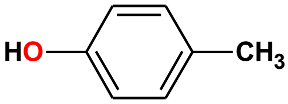<br>***p*-Cresol**<br>4-Methylphenol<br>4-Hydroxytoluene
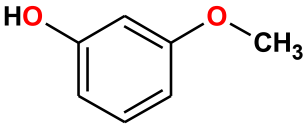<br>**3-Methoxyphenol**<br>*m*-Methoxyphenol<br>3-Hydroxyanisole	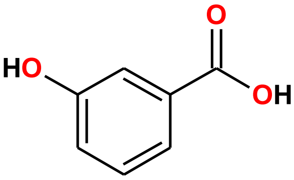<br>**3-Hydroxybenzoic acid**<br>*m*-Salicylic acid<br>3-Carboxyphenol	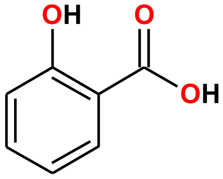<br>**Salicylic acid**<br>2-Hydroxybenzoic acid<br>2-Carboxyphenol
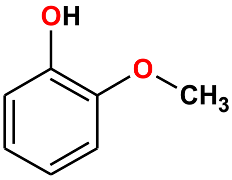<br>**Guaiacol**<br>2-Methoxyphenol<br>2-Hydroxyanisole	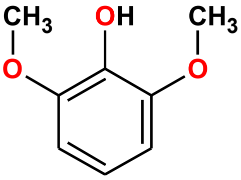<br>**2,6-Dimethoxyphenol**<br>Syringol<br>Pyrogallol 1,3-dimethyl ether	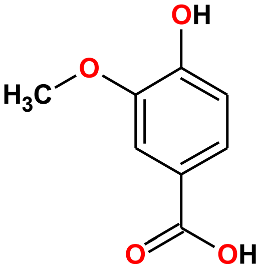<br>**Vanillic acid**<br>4-Hydroxy-3-methoxybenzoic acid
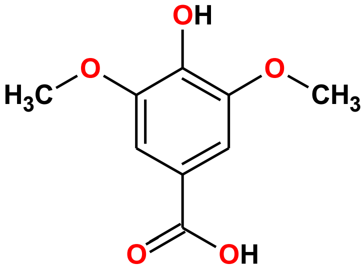<br>**Syringic acid**<br>4-Hydroxy-3,5-dimethoxybenzoic acid<br>Gallic acid 3,5-dimethyl ether		

Blocked phenols

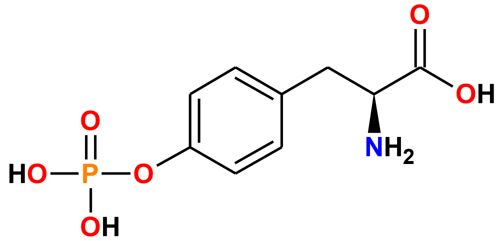

O-Phospho-L-tyrosine
Phosphonotyrosine

Catechols

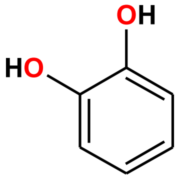<br>**Catechol**<br>Pyrocatechol<br>1,2-Dihydroxybenzene	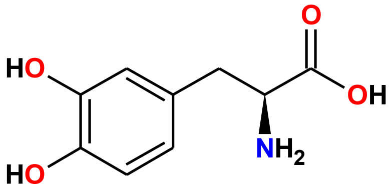<br>**Levodopa**<br>L-DOPA<br>3,4-Dihydroxy-L-phenylalanine	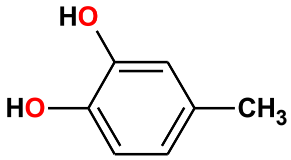<br>**4-Methylcatechol**<br>4-Methylbenzene-1,2-diol<br>3,4-Dihydroxytoluene
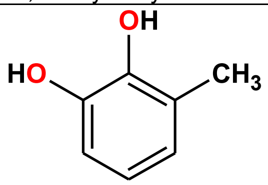<br>**3-Methylcatechol**<br>3-Methylbenzene-1,2-diol<br>2,3-Dihydroxytoluene	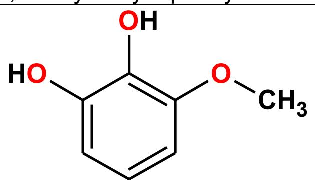<br>**3-Methoxycatechol**<br>3-Methoxypyrocatechol<br>2,3-Dihydroxyanisole	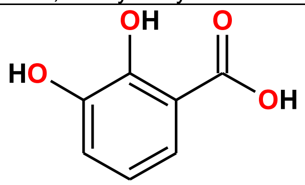<br>**2,3-Dihydroxybenzoic acid**<br>Pyrocatechuic acid<br>Catechol-3-carboxylic acid
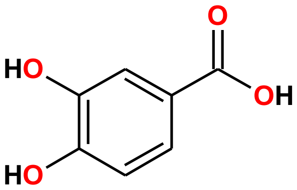<br>**3,4-Dihydroxybenzoic acid**<br>Protocatechuic acid		

o-Aminophenols

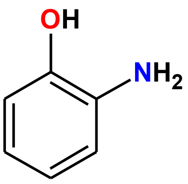<br>**2-Aminophenol**<br>*o*-Aminophenol<br>*o*-Hydroxyaniline	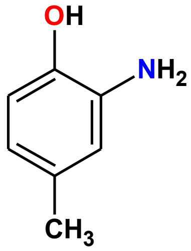<br>**2-Amino-4-methylphenol**<br>2-Amino-*p*-cresol<br>3-Amino-4-hydroxytoluene	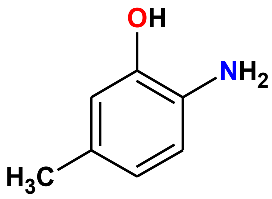<br>**2-Amino-5-methylphenol**<br>2-Hydroxy-4-methylaniline<br>4-Amino-3-hydroxytoluene
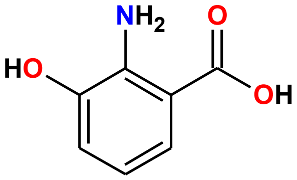<br>**3-Hydroxyanthranilic acid**<br>2-Amino-3-hydroxybenzoic acid	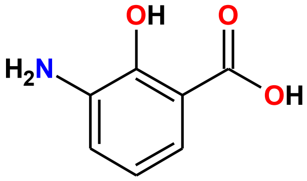<br>**3-Aminosalicylic acid**<br>3-Amino-2-hydroxybenzoic acid	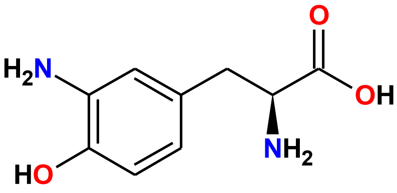<br>**3-Amino-L-tyrosine**<br>3-Aminotyrosine

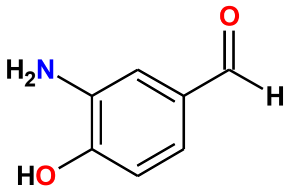<br>**3-Amino-4-hydroxybenzaldehyde**	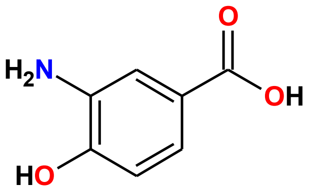<br>**3-Amino-4-hydroxybenzoic acid**	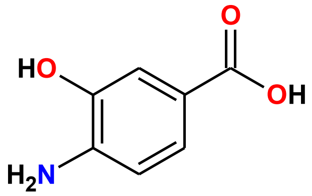<br>**4-Amino-3-hydroxybenzoic acid**

	**Anilines**	
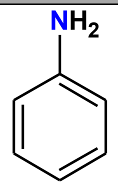<br>**Aniline**<br>Benzenamine<br>Aminobenzene	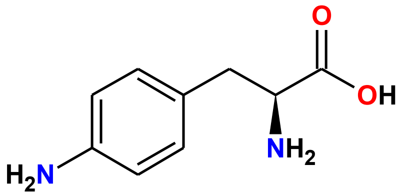<br>**4-Amino-L-phenylalanine**<br>*p*-Aminophenylalanine	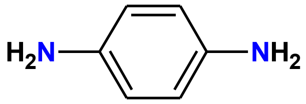<br>***p*-Phenylenediamine**<br>1,4-Diaminobenzene<br>4-Aminoaniline
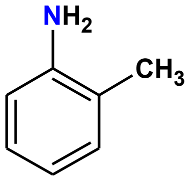<br>***o*-Toluidine**<br>2-Methylaniline<br>2-Aminotoluene	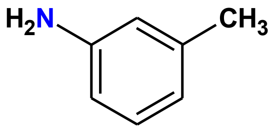<br>***m*-Toluidine**<br>3-Methylaniline<br>3-Aminotoluene	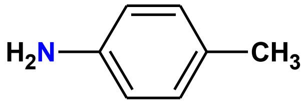<br>***p*-Toluidine**<br>4-Methylaniline<br>4-Methylbenzenamine
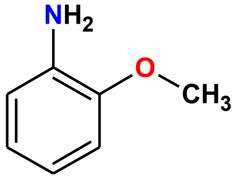<br>***o*-Anisidine**<br>2-Methoxyaniline<br>2-Aminoanisole	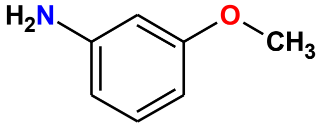<br>***m*-Anisidine**<br>3-Methoxyaniline<br>3-Aminoanisole	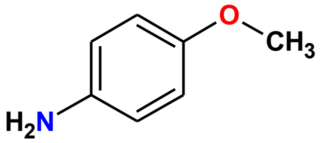<br>***p*-Anisidine**<br>4-Methoxyaniline<br>4-Aminoanisole
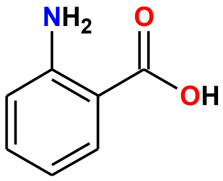<br>**Anthranilic acid**<br>2-Aminobenzoic acid<br>Carboxyaniline	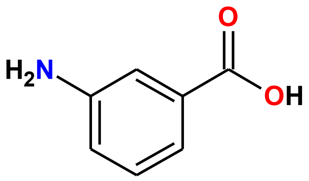<br>**3-Aminobenzoic acid**<br>*m*-Carboxyaniline	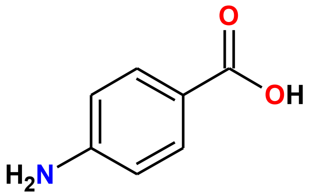<br>**4-Aminobenzoic acid**<br>4-Carboxyaniline
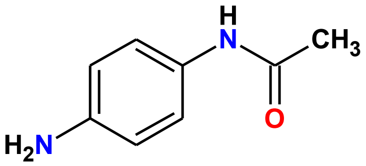<br>**4-Aminoacetanilide**<br>*p*-Aminoacetanilide<br>N-Acetyl-*p*-phenylenediamine		

o-Diaminobenzenes		
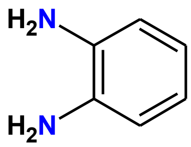<br>***o*-Phenylendiamine**<br>1,2-Diaminobenzene<br>2-Aminoaniline	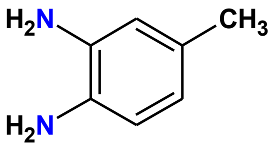<br>**3,4-Diaminotoluene**<br>4-Methyl-*o*-phenylenediamine	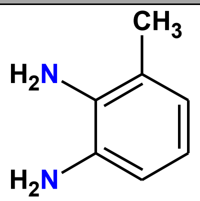<br>**2,3-Diaminotoluene**<br>Toluene-2,3-diamine<br>2-Amino-3-methylphenylamine
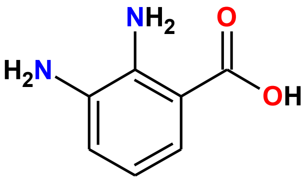<br>**2,3-Diaminobenzoic acid**	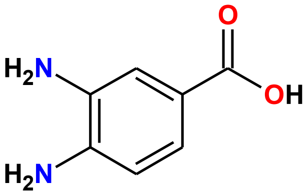<br>**3,4-Diaminobenzoic acid**<br>4-Carboxy-*o*-phenylenediamine	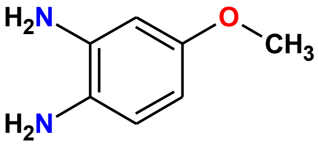<br>**3,4-Diaminoanisole**<br>4-Methoxy-*o*-phenylenediamine<br>4-Methoxy-1,2-phenylendiamine

Aromatic hydrazines		
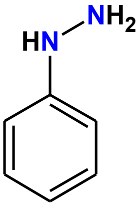<br>**Phenylhydrazine**<br>Hydrazinobenzene	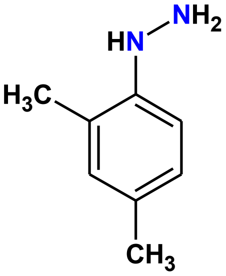<br>**(2,4-Dimethylphenyl)hydrazine**<br>2,4-Dimethylphenylhydrazine<br>2,4-Xylylhydrazine	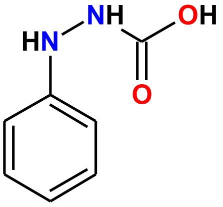<br>**1-Acetyl-2-phenylhydrazine**<br>Acetylphenylhydrazine<br>Hydracetin

Fig. S2. Structures of the TYR-substrates mentioned in the text.
Phenols (including one structures where the phenolic hydroxy group is blocked as a phosphoester), catechols, *ortho*-aminophenols, aromatic mono- and *ortho*-diamines as well as aromatic hydrazine derivates are accepted by TYRs as substrates.

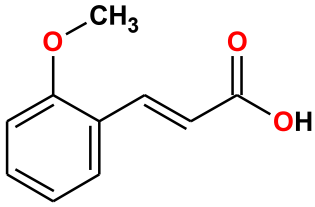<br>**2-Methoxycinnamic acid**<br>(E)-3-(2-Methoxyphenyl)-2-propenoic acid	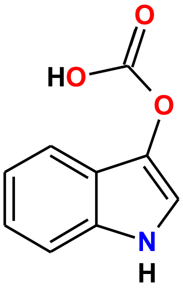<br>**Indole-3-carbonic acid**	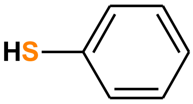<br>**Benzenethiol**<br>Thiophenol<br>Mercaptobenzene

Fig. S3. Structures of three potential TYR-substrates.

(A)

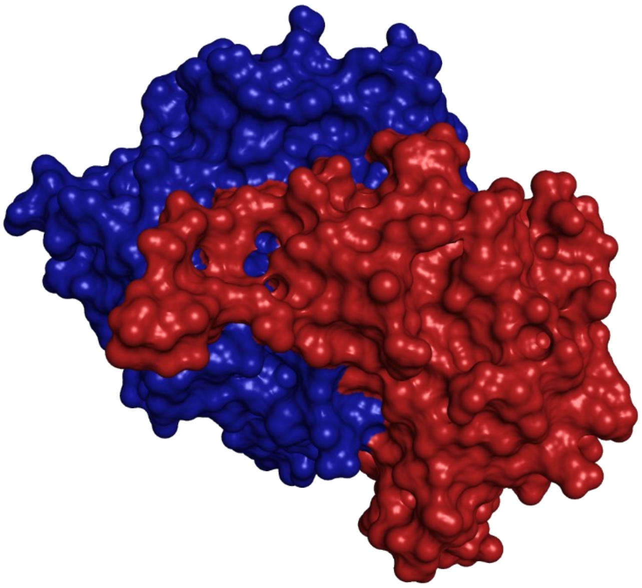

(B)

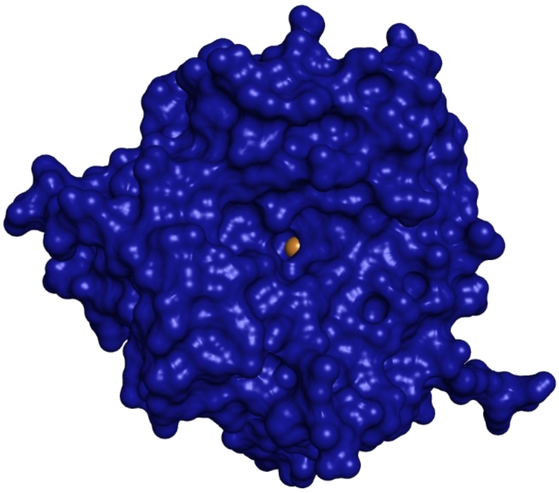

(C)

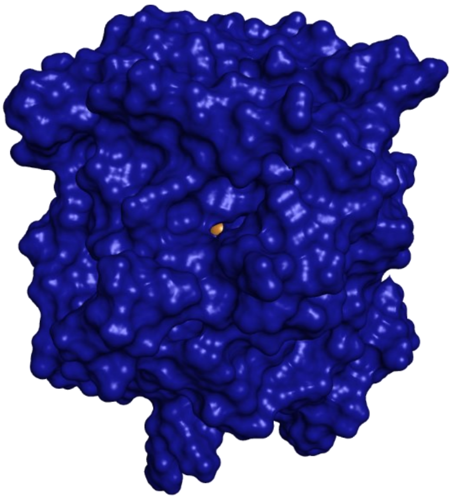

(D)

Feature	Long fungal TYRs	Short fungal TYRs
Amino acids	≈ 500 - 700	≈ 300 - 450
Main domain	Yes	Yes
C-terminal domain	Yes	No
Signal sequence§	Yes / No	Yes
Thioether bond*	Yes	No
His$_{A2}$ - His$_{A1}$#	≥ 20	7

§........ presence of a N-terminal signal sequence targeting the protein to a subcellular organelle or out of the cell

*........ presence of a thioether bond at the 2nd copper-coordinating histidine in the CuA site

#........ number of amino acids between the 1st and 2nd copper-coordinating histidines of the CuA site

Fig. S4. Domain architecture of short fungal tyrosinases.
(A) Crystal structure of the fungal TYR *Ab*PPO4 from the common button mushroom *Agaricus bisporus* (PDB-ID 4OUA)[39]; The main tyrosinase domain that contains the type III copper active site is shown in blue while the C-terminal domain that covers the active site and hinders substrate access is drawn in red. **(B)** depicts *Ab*PPO after proteolytic removal of the C-terminal domain[2] ("active" or "mature" form[3]). The brown sphere visible at the center of the enzyme represents one of the two copper ions of the active center (CuB). The other copper ion (CuA) is located left to it but is concealed by amino acids at the entrance to the active site. **(C)** shows the fungal short tyrosinase *Ao*CO4 from the kōji mold *Aspergillus oryzae* (PDB-ID 5OR3)[40] that does not possess a C-terminal domain. The barely visible brown sphere indicates the position of CuB in the active site. **(D)** Differences in protein architecture between the typical "long" fungal TYRs and short fungal TYRs[25].

Table S1. Kinetic efficiencies of *Nc*TYR on *o*-aminophenols, anilines and the corresponding catechols or phenols[17].

***o*-Aminophenol**	k_{cat} K_M^{-1} / (s^{-1} mM^{-1})	**Catechol**	k_{cat} K_M^{-1} / (s^{-1} mM^{-1})
2-amino-3-hydroxybenzoic acid	0.36 ± 0.053	2,3-dihydroxybenzoic acid	0.055 ± 0.0035
3-amino-2-hydroxybenzoic acid	0.0354 ± 0.00094		
3-amino-4-hydroxybenzoic acid	0.184 ± 0.0047	3,4-dihydroxybenzoic acid	19 ± 0.71
4-amino-3-hydroxybenzoic acid	0.136 ± 0.0038		
**Aromatic *o*-diamine**	k_{cat} K_M^{-1} / (s^{-1} mM^{-1})	**Catechol**	k_{cat} K_M^{-1} / (s^{-1} mM^{-1})
3,4-diaminobenzoic acid	0.29 ± 0.014	3,4-dihydroxybenzoic acid	19 ± 0.71
**Aniline**	k_{cat} K_M^{-1} / (s^{-1} mM^{-1})	**Phenol**	k_{cat} K_M^{-1} / (s^{-1} mM^{-1})
4-aminophenylalanine	0.135 ± 0.0091	4-hydroxyphenylalanine (tyrosine)	340 ± 20
4-aminotoluene (*p*-toluidine)	0.23 ± 0.045	4-hydroxytoluene (*p*-cresol)	320 ± 50
4-aminoacetanilide	0.18 ± 0.023	4-hydroxyacetanilide	45 ± 2.6

Reaction conditions: 0.1 M potassium phosphate buffer pH 7 (3 ml), substrate concentration range 0.24-24 mM; temperature 24 °C.

Table S2. Kinetic efficiencies of *Ab*T on *o*-aminophenols, anilines and the corresponding catechols or phenols[23].

***o*-Aminophenol**	k_{cat} K_M^{-1} / (s^{-1} mM^{-1})	**Catechol**	k_{cat} K_M^{-1} / (s^{-1} mM^{-1})
2-amino-3-hydroxybenzoic acid	0.057 ± 0.0071	2,3-dihydroxybenzoic acid	0.36 ± 0.058
3-amino-2-hydroxybenzoic acid	0.36 ± 0.049		
3-amino-4-hydroxybenzoic acid	4.1 ± 0.38	3,4-dihydroxybenzoic acid	120 ± 17
4-amino-3-hydroxybenzoic acid	3.3 ± 0.23		
3-amino-4-hydroxytoluene	11 ± 2.3	4-methylcatechol	8400 ± 880
4-amino-3-hydroxytoluene	11 ± 2.2		
3-amino-L-tyrosine	1.5 ± 0.34	L-DOPA	190 ± 44
2-aminophenol	78 ± 8.8	catechol	5500 ± 390
**Aromatic *o*-diamine**	k_{cat} K_M^{-1} / (s^{-1} mM^{-1})	**Catechol**	k_{cat} K_M^{-1} / (s^{-1} mM^{-1})
2,3-diaminobenzoic acid	0.18 ± 0.044	2,3-dihydroxybenzoic acid	0.36 ± 0.058
3,4-diaminobenzoic acid	0.53 ± 0.097	3,4-dihydroxybenzoic acid	120 ± 17
2,3-diaminotoluene	0.11 ± 0.017	3-methylcatechol	90 ± 13
3,4-diaminotoluene	9 ± 1.6	4-methylcatechol	8400 ± 880
4-methoxy-1,2-phenylendiamine	4.2 ± 0.69	3-methoxycatechol	14 ± 1.5
1,2-diaminobenzene	1.2 ± 0.17	catechol	5500 ± 390
**Aniline**	k_{cat} K_M^{-1} / (s^{-1} mM^{-1})	**Phenol**	k_{cat} K_M^{-1} / (s^{-1} mM^{-1})
aniline	0.7 ± 0.11	phenol	18 ± 2.7
2-aminobenzoic acid	0.000055 ± 0.000009	2-hydroxybenzoic acid	0.018 ± 0.0014
3-aminobenzoic acid	0.00118 ± 0.000091	3-hydroxybenzoic acid	0.27 ± 0.039
4-aminobenzoic acid	0.036 ± 0.0034	4-hydroxybenzoic acid	8 ± 1.1
2-aminotoluene	0.11 ± 0.016	2-methylphenol	5 ± 0.66
3-aminotoluene	0.37 ± 0.038	3-methylphenol	12 ± 2
4-aminotoluene (*p*-toluidine)	1.0 ± 0.12	4-methylphenol (*p*-cresol)	42 ± 6.9
2-aminoanisole	0.35 ± 0.055	2-hydroxyanisole	1.0 ± 0.12
3-aminoanisole	0.8 ± 0.13	3-hydroxyanisole	19 ± 2.1
4-aminoanisole	14 ± 2.8	4-hydroxyanisole	2300 ± 300
4-aminophenylalanine	0.02 ± 0.024	4-hydroxyphenylalanine (tyrosine)	30 ± 6.3

Reaction conditions: 0.06-0.9 µM *Ab*T in 30 mM sodium phosphate buffer (pH 7.0) with suitable concentrations of either the reductant ascorbic acid or reduced nicotinamide adenine dinucleotide (NADH), the nucleophile 3-methyl-2-benzothiazolinonehydrazone (MBTH) or no reagent addition; temperature 25 °C.

Author Contributions

M.P. Conceptualization: Lead; Data curation: Lead; Writing - Original Draft: Lead; Writing - Review & Editing: Lead.

A.R. Conceptualization: Supporting; Funding acquisition: Lead; Project administration: Lead; Supervision: Lead; Writing - Original Draft: Supporting; Writing - Review & Editing: Supporting.
